# Simulating Costs of Intravenous Biosimilar Trastuzumab vs. Subcutaneous Reference Trastuzumab in Adjuvant HER2-Positive Breast Cancer: A Belgian Case Study

**DOI:** 10.3390/ph14050450

**Published:** 2021-05-11

**Authors:** Steven Simoens, Arnold G. Vulto, Pieter Dylst

**Affiliations:** 1 Department of Pharmaceutical and Pharmacological Sciences, KU Leuven, 3000 Leuven, Belgium; a.vulto@gmail.com; 2 Hospital Pharmacy, Erasmus University Medical Center, 3000 CA Rotterdam, The Netherlands; 3 EG nv/sa, 1020 Brussels, Belgium; pieter.dylst@eg.be

**Keywords:** trastuzumab, biosimilar, intravenous, subcutaneous, HER2-positive breast cancer, drug costs, healthcare costs, cost simulation

## Abstract

This study aimed to compare drug costs and healthcare costs of a 1 year adjuvant course with intravenous biosimilar trastuzumab vs. subcutaneous reference trastuzumab in HER2-positive breast cancer from the Belgian hospital perspective. Our simulation is based on the methodology used by Tjalma and colleagues, and considered costs of drugs, healthcare professional time and consumables. We calculated intravenous drug costs for different body weights, and computed drug costs and healthcare costs to treat 100 patients with either trastuzumab formulation, assuming a binomial body weight distribution in this sample. Scenarios were run to account for drug discounts and intravenous vial sharing. Drug costs amounted to €1,431,282 with intravenous biosimilar trastuzumab and €1,522,809 with subcutaneous reference trastuzumab for a sample of 100 patients in the base case analysis. When healthcare professional time and consumables were also considered, healthcare costs with intravenous biosimilar trastuzumab were similar to those with subcutaneous reference trastuzumab. Differences in healthcare costs between intravenous biosimilar trastuzumab and subcutaneous reference trastuzumab depended on the level of discounts on these formulations and on intravenous vial sharing. Our case study demonstrates that comparing costs of intravenous vs. subcutaneous formulations is complex and multifactorial, and entails more than a simple cost comparison of products.

## 1. Introduction

Trastuzumab has played, and continues to play, a pivotal role in the standard first-line treatment of HER2-positive breast cancer for approximately two decades. Initial approval was based on the significant overall survival advantage demonstrated in key clinical trials in both the metastatic [1,2,3] and adjuvant [4,5] breast cancer settings. Until relatively recently, trastuzumab was administered using intravenous (IV) regimens either as monotherapy or, more usually, in combination with chemotherapy or biologic therapy. A subcutaneous (SC) formulation of trastuzumab was subsequently developed and was approved for use in Europe. The IV and SC formulations of trastuzumab show comparable pharmacokinetics [6,7,8], and have been reported to have equivalent (non-inferior) efficacy and tolerability in the HannaH, PrefHer and MetaspHer clinical studies [9,10,11]. In 2020, the global ex-factory turnover of reference trastuzumab accounted for more than US$4 billion [12].

A drug cost comparison at 2017 ex-factory prices in Belgium has been performed for the IV and SC formulations of reference trastuzumab for patients in different weight categories [13]. The calculation for a total of 18 cycles of adjuvant trastuzumab showed higher drug costs with the SC formulation for patients weighing >75 kg and with the IV formulation for those weighing <75 kg. The main reason for this was the single fixed available dose for the SC formulation (600 mg).

A biosimilar is a biological medicine that is highly similar to another already approved biological medicine (the “reference medicine”) and does not show clinically meaningful differences from the reference medicine with respect to pharmaceutical quality, efficacy, and safety [14]. Several IV trastuzumab biosimilars have reached advanced stages of clinical development globally [15], some of which are available in Europe.

The aim of this case study was to compare drug costs and healthcare costs of IV biosimilar trastuzumab vs. SC reference trastuzumab (Herceptin^®^, Roche) as adjuvant treatment for one year in women with HER2-positive breast cancer from the hospital perspective in Belgium as an example to show the multifactorial character of an at-first-sight simple comparison. Our study is based on the methodology used by Tjalma and colleagues [13,16].

## 2. Results

**Figure 1:**
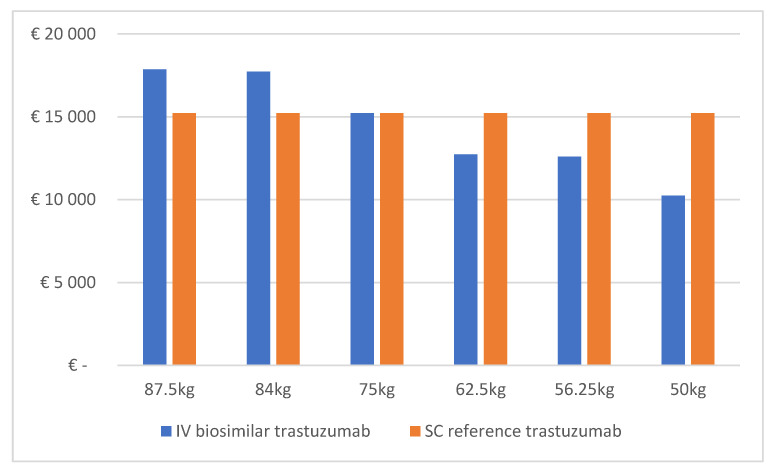
Drug costs for 1 year course of adjuvant treatment with IV biosimilar trastuzumab or with SC reference trastuzumab.

Drug costs for a 1 year course of adjuvant treatment with IV biosimilar trastuzumab (at 2020 Belgian list prices) ranged from €17,858 for a patient weighing 87.5 kg to €10,244 for a patient weighing 50 kg (see Figure 1). In the case of a 1 year course with SC reference trastuzumab, drug costs amounted to €15,228, irrespective of patient body weight. Thus, treatment with IV biosimilar trastuzumab was less expensive in terms of drug costs than with SC reference trastuzumab for patients weighing up to 75 kg (see Figure 1).

**Figure 2:**
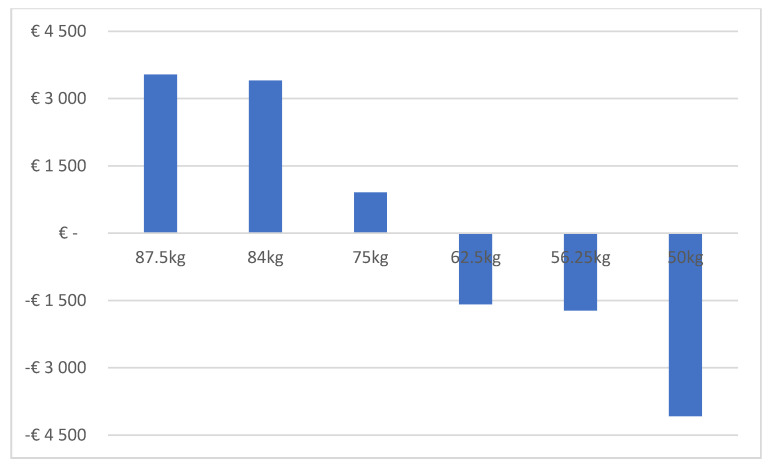
Difference in healthcare costs of 1 year course of adjuvant treatment with IV biosimilar trastuzumab as compared with SC reference trastuzumab.

We next determined the difference in healthcare costs (i.e., drug costs, healthcare professional time costs and consumables costs) between the IV and SC formulations. This calculation took into account that the IV trastuzumab administration was previously estimated to cost €907.20 per course more than SC administration in terms of healthcare professional time costs and consumables costs [16]. Figure 2 shows that healthcare costs for a 1 year course of adjuvant treatment with IV biosimilar trastuzumab were lower than costs with SC reference trastuzumab for a patient weighing 50 kg, for a patient weighing 56.25 kg and for a patient weighing 62.5 kg. Healthcare costs with IV biosimilar trastuzumab exceeded those with SC reference trastuzumab for a patient weighing 75 kg, for a patient weighing 84 kg and for a patient weighing 87.5 kg; the reason being that IV trastuzumab is dosed on a mg/kg basis and the SC formulation has a fixed dose for all body weights.

When calculated for a sample of 100 patients, the difference in drug costs between the IV and SC formulations amounted to €91,527 (see Table 1). When also considering healthcare professional time and consumables, healthcare costs for a 1 year course of adjuvant treatment with IV biosimilar trastuzumab were similar to those with SC reference trastuzumab (i.e., savings of €807 with IV biosimilar trastuzumab). Furthermore, Table 1 shows that differences in healthcare costs between IV biosimilar trastuzumab and SC reference trastuzumab depended on the level of discounts on these formulations. In a scenario assuming a discount of 50% on IV biosimilar trastuzumab and 20% on SC reference trastuzumab, savings in healthcare costs of €411,886 were generated by treating 100 patients with IV biosimilar trastuzumab as compared to SC reference trastuzumab. These savings increased to €430,192 when IV vial sharing is considered.

## 3. Discussion

This study has simulated drug costs and healthcare costs for a 1 year course of adjuvant treatment with either IV biosimilar trastuzumab or SC reference trastuzumab in HER2-positive breast cancer patients in Belgium. Our results indicated that the cost difference between IV and SC formulations depends on patient body weight, drug discounts and IV vial sharing.

In our base case analysis, drug costs were less for IV biosimilar trastuzumab for a patients weighing less than 75 kg. The median weight of women with breast cancer is invariably <75 kg and has ranged from 64 to 72 kg in European studies comparing IV and SC reference trastuzumab administration [17,18,19,20]. Therefore, it can be expected that drug costs of IV biosimilar trastuzumab would be lower than for SC reference trastuzumab for the majority of patients.

When considering healthcare costs, our base case analysis took into account that IV administration is associated with more costs related to healthcare professional time and consumables than SC administration, in addition to differences in drug costs. However, savings in healthcare professional time and consumables with SC administration might not be as high when trastuzumab is given in combination with chemotherapy. When trastuzumab is administered in combination with chemotherapy, this is usually for the first 6–8 cycles of 18 cycles during adjuvant therapy. During these 6–8 cycles, there are potential cost savings with respect to healthcare professional time and consumables with IV trastuzumab administration by piggy backing on the costs that must be applied for IV chemotherapy administration during concurrent or sequential administration. The combination of trastuzumab with chemotherapy is usual practice (94%) during adjuvant therapy across German hospitals [21], whereas trastuzumab monotherapy is the norm in the Southeast Netherlands (100%) [22] and most common in Southeast Wales (83%) [23].

Multiple studies have reported that SC reference trastuzumab administration is associated with less indirect costs related to productivity loss than IV administration [16,19,20]. Our analysis did not consider productivity loss and, hence, underestimated savings of SC vs. IV trastuzumab administration. However, such indirect costs associated with trastuzumab administration (irrespective of administration route) are relatively low (1–4%) when compared to total costs [24].

When we applied healthcare cost estimates to a sample of 100 patients, lower drug costs with IV biosimilar trastuzumab as compared to SC reference trastuzumab offset higher costs of healthcare professional time and consumables in our base case analysis. Also, we ran scenario analyses accounting for drug discounts and for the re-use of IV vial leftovers. We believe that these scenarios more accurately reflect market and clinical practices in Belgium, even though the related input parameters are associated with more uncertainty and resulting cost difference estimates are illustrative rather than exact. In terms of generalizability to other healthcare systems, healthcare cost differences between these trastuzumab formulations of course depend on the difference between the drug procurement cost and reimbursement rate, on local healthcare professional and consumable costs, and on the hospital or retail setting in which IV and SC formulations are typically provided.

Our results are in line with those of an Italian study [25], which found that treatment with IV biosimilar trastuzumab was less expensive than with SC reference trastuzumab in patients weighing less than a specific threshold. Also, this study corroborated our finding that, when vial leftovers are used for other patients, savings with IV biosimilar trastuzumab grew.

We hope that our case study contributes to a more differentiated view on the difference between IV and SC formulations beyond the bare price of the products alone. Indeed, we acknowledge that other factors may also play important roles like the business models of hospitals and the earning system of physicians. A hospital that is short in IV administration capacity, and gains limited earnings from IV administrations, may like to avoid investments to expand such (expensive) capacity. On the other hand, if physician reimbursement for IV administration is higher than for SC administration, then it will be attractive for physicians to favor the former. In a number of countries, parenteral drugs are increasingly being administered outside the hospital, closer to where patients are living. Such initiatives are more dependent on the availability of SC formulations.

There are a number of limitations in our study. The estimate of cost savings related to healthcare professional time and consumables with SC trastuzumab administration related to 2017 [16], while drug prices related to 2020. Although the former are likely to have increased since then, this is unlikely to change our result that healthcare cost differences between IV and SC trastuzumab formulations depend on patient body weight. Also, any analysis is dependent on the potential for changing prices and discounts that might be offered in particular situations for both IV biosimilar trastuzumab and SC reference trastuzumab, as underlined by our sensitivity analysis.

Few studies have explored cost differences between IV biosimilar trastuzumab and SC reference trastuzumab [26]. More research is required that replicates our cost estimates in healthcare systems that are organized and financed differently than in Belgium and that takes into account market dynamics and shifts in prescribing practices between different trastuzumab formulations.

## 4. Materials and Methods

Calculations of drug costs for IV biosimilar trastuzumab vs. SC reference trastuzumab were conducted in the same manner and following the same methods as reported for the comparison of IV vs. SC reference trastuzumab in the study by Tjalma and colleagues [13]. Drug costs were compared for a 1 year trastuzumab course in the adjuvant HER2-positive breast cancer setting in Belgium. For IV biosimilar trastuzumab, there is an initial loading dose of 8 mg/kg infused over 90 min, followed by maintenance doses of 6 mg/kg infused over 30 min every 3 weeks for a total of 18 cycles. For SC reference trastuzumab, the equivalent schedule of 600 mg SC is administered by slow injection over 2–5 min every 3 weeks for 18 cycles. For each treatment (IV biosimilar vs. SC reference), the number of vials required per patient was determined for different patient body weights (87.5, 84, 75, 62.5, 56.25 and 50 kg) and was rounded to the next highest half vial (as is usual practice). The number of vials was then multiplied by the ex-factory list price in 2020 to calculate drug costs. List prices were reduced by 15% given that Belgian hospitals can only invoice 85% of a drug’s list price to the National Institute for Health and Disability Insurance once a biosimilar is available [27]. All prices were exclusive of tax. The 85% list price of IV biosimilar trastuzumab (Herzuma^®^) was €276.87 per 150 mg vial and that for SC reference trastuzumab (Herceptin^®^) was €846.01 per 600 mg vial [28].

Next, we compared healthcare costs for IV biosimilar trastuzumab vs. SC reference trastuzumab at the previously defined different patient body weights (see above) by taking into consideration potential savings through SC use that have been previously estimated by Tjalma and colleagues [16]. They estimated savings at 2017 prices of SC vs. IV administration of €907.20 per course related to healthcare professional (i.e., nurse, pharmacist and assistant) and consumables (e.g., syringes, needles, alcohol, swabs, etc.) costs. Oncologist time was not included as a healthcare professional cost as this consultation visit was assumed to be the same for both the IV and SC reference formulations.

Drug costs and healthcare costs to treat 100 patients with either trastuzumab formulation were then calculated assuming the following numbers of patients in each body weight category: 87.5 kg (*n* = 7); 84.0 kg (*n* = 16); 75.0 kg (*n* = 25); 62.5 kg (*n* = 25); 56.25 kg (*n* = 20); and 50.0 kg (*n* = 7). This distribution of patients by body weight category was based on the binomial distribution normally found among patients with early-stage HER2-positive breast cancer [17,18,19,20].

In addition to the base case analysis, we conducted a sensitivity analysis that accounts for discounts offered by the manufacturer to the hospital. As discounts are confidential, we ran multiple scenarios, but the scenario assuming a discount of 50% on the IV biosimilar formulation and 20% on the SC reference formulation was deemed most realistic after consultation with an industry expert.

The base case analysis used an IV vial (or half a vial for IV trastuzumab in Belgium) as the unit of measurement. Hence, costs associated with the total number of vials administered over 18 cycles were calculated, even if some of the last vial’s contents had to be discarded. However, in clinical practice, any drug not used may not necessarily be wasted but rather used for other patients scheduled for treatment in parallel on the same day [20]. This practice is common in many countries [24] and also appears to be the practice in Belgian hospitals. If hospitals use the potentially wasted drug in other patients, it will generate savings from the hospital perspective. Therefore, we ran a second scenario in which cost estimates accounted for discounts and reflected actual use of the IV biosimilar formulation (i.e., not rounded to the next half vial).

All calculations were performed in Microsoft Excel 2016.

## Figures and Tables

**Table 1:** Drug costs and healthcare costs of treating 100 patients with IV biosimilar trastuzumab vs. SC reference trastuzumab.

	Base Case	Scenario with 20% Discount on IV Biosimilar and on SC Reference Trastuzumab	Scenario with 35% Discount on IV Biosimilar and 20% Discount on SC Reference Trastuzumab	Scenario with 35% Discount on IV Biosimilar and on SC Reference Trastuzumab	Scenario with 50% Discount on IV Biosimilar and 20% Discount on SC Reference Trastuzumab
*Drug costs*					
IV	€1,431,282	€1,145,026	€930,333	€930,333	€715,641
SC	€1,522,809	€1,218,247	€1,218,247	€989,826	€1,218,247
IV-SC	−€91,527	−€73,222	−€287,914	−€59,493	−€502,606
*Healthcare costs*					
IV-SC	−€807	€17,498	−€197,194	€31,227	−€411,886

## Data Availability

Access to data supporting reported results can be requested from the corresponding author.
